# Dual-task and electrophysiological markers of executive cognitive processing in older adult gait and fall-risk

**DOI:** 10.3389/fnhum.2015.00200

**Published:** 2015-04-17

**Authors:** Elizabeth A. Walshe, Matthew R. Patterson, Seán Commins, Richard A. P. Roche

**Affiliations:** ^1^Department of Psychology, Maynooth UniversityMaynooth, Kildare, Ireland; ^2^Insight Center for Data Analytics, University College DublinDublin, Ireland

**Keywords:** gait, falls, dual-task, executive function, ERP, aging

## Abstract

The role of cognition is becoming increasingly central to our understanding of the complexity of walking gait. In particular, higher-level executive functions are suggested to play a key role in gait and fall-risk, but the specific underlying neurocognitive processes remain unclear. Here, we report two experiments which investigated the cognitive and neural processes underlying older adult gait and falls. Experiment 1 employed a dual-task (DT) paradigm in young and older adults, to assess the relative effects of higher-level executive function tasks (n-Back, Serial Subtraction and visuo-spatial Clock task) in comparison to non-executive distracter tasks (motor response task and alphabet recitation) on gait. All DTs elicited changes in gait for both young and older adults, relative to baseline walking. Significantly greater DT costs were observed for the executive tasks in the older adult group. Experiment 2 compared normal walking gait, seated cognitive performances and concurrent event-related brain potentials (ERPs) in healthy young and older adults, to older adult fallers. No significant differences in cognitive performances were found between fallers and non-fallers. However, an initial late-positivity, considered a potential early P3a, was evident on the Stroop task for older non-fallers, which was notably absent in older fallers. We argue that executive control functions play a prominent role in walking and gait, but the use of neurocognitive processes as a predictor of fall-risk needs further investigation.

## Introduction

The ability to walk while multitasking, to negotiate terrains and obstacles, all while attending to environmental sensory distractions, distinguishes walking gait and navigation as highly complex behaviors (Hausdorff et al., 2005). Falls while walking are a common problem for older adults, globally, with approximately 50% of adults over the age of 80 falling each year (World Health Organization, 2008). Consideration of top-down processing mediating gait has grown with increasing evidence of a cognitive-motor link (for reviews see Woollacott and Shumway-Cook, 2002; Yogev-Seligmann et al., 2008).

However, cognition is not a unitary construct, but rather a complex system of multiple and varied processes (internal monitoring and external responding; Al-Yahya et al., 2011). Thus far, the neurocognitive processes underlying gait and falls remain unclear. This paper presents two experiments to address two different, but related gaps in the literature on the role of the cognitive-motor link in gait and fall research: (1) Which higher-level cognitive function is most utilized or relied upon during gait performance in healthy older adults?; and (2) Can neural electrophysiological markers of higher-level cognitive impairment be found in older fallers compared to older non-faller adults?

Experiment 1 employs a controlled, comparative dual-task (DT) paradigm to probe the respective effects of a variety of secondary higher-level cognitive tasks on young and older adult gait. The DT paradigm is commonly used to investigate the overlap in processing during cognitive tasks and walking gait (Plummer-D’Amato et al., 2008; Al-Yahya et al., 2011). A systematic review and meta-analysis of the cognitive-motor DT literature has suggested that higher-level executive function processes affect gait more than mere distraction of attention during walking (Hausdorff et al., 2005; Al-Yahya et al., 2011). Within an Irish older adult sample, neuropsychological tests of processing speed, short-term memory and sustained attention contributed to slower gait speed on both single and dual gait tasks, with an additional specific role of executive function for the DT, but not single-task performance (Killane et al., 2014). Executive function and attention (but not visual-spatial, memory or global cognition) have also been shown to correlate with, and prospectively predict falls in undiagnosed older adults (Mirelman et al., 2012; see also Holtzer et al., 2005; Herman et al., 2010; Buracchio et al., 2011).

The DT review by Al-Yahya et al. (2011) also highlighted a large problem of methodological variability in the DT literature, which has ramifications for translation to the clinical setting. Previous studies have often employed only one executive function DT, or have failed to include non-executive tasks for relative comparisons (van Iersel et al., 2008). The overuse of general, non-specific cognitive or distracter tasks, and the broad variability in the choice of target task (memory recall tasks, motor tray carrying task, spontaneous speech) have contributed to ongoing ambiguity regarding the relative contribution of specific higher-level processes to walking. Consequently, preliminary clinical screening and DT training studies have yielded mixed efficacy (Plummer-D’Amato et al., 2012; Taylor-Piliae et al., 2012). For example, Plummer-D’Amato et al. (2008) found that once weekly DT training had no greater effect on gait outcomes than balance training alone. However, the DTs used were not domain-specific, with no clear executive cognition components (spontaneous speech, alphabet recitation and a coin transfer task). To address these shortcomings and the findings of Al-Yaha’s review, we use domain-specific tasks to compare different executive functions with non-executive motor and verbal responding tasks.

One way to further identify and elucidate the contributions of specific higher-level cognitive processes to gait is with the use of neuroimaging and physiological recording approaches. Fall-related executive cognitive decline, slower gait speed and DT gait capacity have been associated with differential functioning and structural changes in frontal areas of the brain (Gunning-Dixon and Raz, 2003; Harada et al., 2009; Holtzer et al., 2011; Rosano et al., 2012; Meester et al., 2014). Stimulation to the left dorsolateral prefrontal cortex (DLPFC) using transcranial direct current stimulation (tDCS) has also been shown to improve postural control and reduce changes in gait during DT walking in healthy young adults (Zhou et al., 2014). Frontal cortical structures such as the prefrontal cortex (PFC), anterior cingulated cortex (ACC), and also the posterior parietal cortex (PPC), are associated with higher-level cognitive control (Kim et al., 2013; Tang et al., 2013).

Increasingly, changes in frontal neuroelectrical activation, and specifically the P300 waveform, have been associated with aging and frontal attention/executive function (Polich, 2007; O’Connell et al., 2012). While there is little research comparing electroencephalogram (EEG)-recorded event related potentials between faller and non-faller older adults, some studies suggests a link between P3 amplitude relating to executive function (inhibition and working memory), and physical activity in older adults (Chang et al., 2013; Fong et al., 2014). Furthermore, a study of visual-spatial attention revealed an association between fall-risk and a greater N1 for poorer discrimination of task-irrelevant stimuli (in the left-field), and a larger P3 amplitude for target processing in low discrimination conditions (Nagamatsu et al., 2013). While wireless EEG recordings during near natural walking and dual-tasking in older adults are still in a preliminary and proof-of-concept stage (Marcar et al., 2014), initial findings in young adults show altered EF-related N2-P3 components during DT walking (De Sanctis et al., 2014).

Taken together, these findings indicate a greater need for cognitive control neural compensation for maintaining a successful normal gait in older age, and for DT walking in both young and older adults. Liu et al. (2014) propose that increased falls in older adults are a consequence of age-related impairments in neural motor outputs, resulting in walking gait becoming more attentionally-demanding, necessitating increased cognitive control. This argument relates to compensation hypotheses of neural aging (Cabeza et al., 2002; Park and Reuter-Lorenz, 2009). Park and Reuter-Lorenz (2009) posit that age-related over-activation of frontal areas suggests compensatory neural recruitment. Within these theoretical frameworks, older-adult fallers (without peripheral physiological impairment such as muscular or skeletal problems) could be considered to lack the plastic reorganization or compensatory over-activation necessary to circumvent age-related cognitive decline.

Experiment 2 aims to establish if event-related potential (ERP) markers of executive impairment can be found in older fallers in contrast to older non-fallers, by comparing working memory and sustained attention/conflict processing performances with gait performance and history of falls status. An n-Back and Stroop task were used to target these two different types of executive functioning (EF) which have been shown to correlate with gait and falls, and greater DT costs (as outlined above: Beurskens and Bock, 2012), and which have previously defined age-related ERP components for comparison (Gajewski and Falkenstein, 2014; Zurrón et al., 2014). While the continued advancement of mobile-EEG recording will surely enhance understanding of the supraspinal activity underlying gait, the current study aimed to utilize accessible and applicable methods which could potentially translate to a clinical setting. If we can identify a specific neuropsychological task or ERP marker of fall-risk in otherwise healthy older adults while seated, this would allow for an accessible and easily administered alternative clinical screening procedure.

The main hypotheses were: (1) there would be subtle gait impairments (slower speed, increased variability) in older adult fallers in comparison to non-fallers and young adults; (2) higher-level EF DTs would affect gait (speed, stride time, variability) comparatively more than basic attention and motor response tasks, with greater effects seen in older adults; (3) age-related and fall-related poorer EF performances would relate to subtle gait impairments; and (4) EF-related later ERP components (N2, P3) would reflect behavioral and gait impairments in fallers compared to non-fallers, and compared to younger adults. More specifically, we want to investigate if P3 amplitude is reduced in older adults (as previously reported by Mager et al., 2007; Zurrón et al., 2014), and more so in older fallers, who may have limited available neural resources for allocation to task demands.

## Experiment 1

### Materials and Methods

For Experiment 1, a sample of 20 healthy young adults aged between 19–28 years (*M* = 21.10 ± 1.80; 50% male) and 17 older adults aged 55+ years (*M* = 61.88 ± 6.23; 35% male) was recruited as volunteers from Maynooth University campus and surrounding locality. Two older adult participants were identified as 52 years of age after recruitment, but their data was retained within the sample as their scores did not unduly influence the data, or fall outside 3 × IQR of the group mean (only extreme outliers at 3 × IQR were removed from the data set, to avoid loss of data). Demographically, it was noted that 6 of the older adults reported experiencing a fall in 12 months previous (aged: 52, 61, 63, 68, 68, and 74). Exclusion criteria were screened by telephone checklist prior to the experiment and included: self-reported or history of clinically diagnosed severe uncorrected sensory impairments; cardiovascular, psychological or neurological impairments (including any diagnosis of MCI or Dementia); any muscular or bone problems likely to cause balance/gait impairments. The Maynooth University Research Ethics Committee approved all experiments, and all participants gave written informed consent at the commencement of their participation.

For comparison between the age groups, the National Adult Reading Test (NART: Nelson, 1982) was used as a measure of pre-morbid intelligence; which correlates with years of education (Crawford et al., 1988). The older adults also completed the Falls Efficacy Scale-International (FES-I: Yardley et al., 2005), the Standardized Mini Mental State Examination (SMMSE: Molloy et al., 1991) and the Montreal Cognitive Assessment (MoCA: Nasreddine et al., 2005). There were no significant differences between the groups on NART scores (full scale IQ: *t*_(35)_ = 1.59, *p* = 0.125), and all older adults had an MMSE score > 28 (*M* = 29.41 ± 0.8) and MoCA score > 23 (*M* = 26.29 ± 1.83). The older adult group reported a mean FES-I score of 25.76 (±7.34) indicating only moderate concern of falling (Delbaere et al., 2010). Following completion of the control measures, participants conducted two separate normal walking trials (single walking task: SWT), seated single cognitive tasks (SCT), and combined walking plus cognitive DT trials. This design allows for the investigation of bi-directional DT effects on both gait and cognitive performance. One normal walking task (SWT) was completed before and after the SCT and DT conditions; the order of which were counter-balanced across participants; half completed seated first, half completed DT first. All tasks were completed in one session lasting approximately 45 min in total (with shorts breaks offered to participants between tasks).

#### Gait Analysis

Participants completed 2 normal walking gait trials, and 5 DT (walking plus secondary task) trials in total, walking along an empty open corridor. Two single-task trials were recorded to obtain an average of usual pace steady state walking. Each trial consisted of walking at a self-selected walking speed (SSWS) along a 20 m walkway four times, with an about-turn at each end. This protocol allowed for enough steady-state gait cycles on each pass, to analyze normal walking gait outside of start/stop and turn-related slowing and acceleration. Gait data was recorded using two wireless inertial measurement sensors (SHIMMER™: Burns et al., 2010) attached to the shank of the left and right leg with Velcro straps. The sensors recorded at a sampling rate of 102.4 Hz, and transmitted tri-axial accelerometer and gyroscope data, via Bluetooth, to a recipient computer for off-line analysis. The inertial sensor data were then processed to calculate stride time using a previously-validated method (Greene et al., 2010). This method is based on finding features in the sagittal plane gyroscope signal from the shank and has been used in previous work analyzing gait performance with the use of inertial sensor technology (Patterson et al., 2014). Gait speed was recorded manually with a stopwatch and video camera.

#### Cognitive Tasks

Five tasks (described below) were used in both seated cognitive trials and DT walking trials. Each task ran for 60 s in both conditions, with accuracy and/or reaction times recorded (depending on the nature of the task). PC-generated auditory stimuli were presented via laptop speakers using E-Prime stimulus presentation software (Psychology Software Tools, Pittsburgh, PA) which automatically recorded accuracy and reaction time data. In the SCT condition, participants sat approximately 104 cm from a blank screen laptop, while the stimuli played through the speakers. In both gait conditions, the participant carried a wireless mouse in their right hand for tasks that required a button-press response. Two tasks required verbal responses without laptop-presented stimuli; these responses were manually recorded (with pen and paper) by the experimenter.

A motor response (Motor) task and alphabet recitation (ABC) task were used as simple attention-demanding control tasks without stimulus differentiation or decision-making components. The Motor task presented a single auditory tone (16-Bit WAV file; 1411 kbps; 1000 ms long with a 3000 ms response window from start of stimulus) at randomly varied delay intervals, (500 ms, 750 ms or 1000 ms). Participants were instructed to quickly respond with a wireless mouse click (held in their right hand). The single-stimulus and single-response option in this task circumvented the need for any stimulus differentiation or decision-making/response selection. The ABC task required participants to recite the alphabet at a self-selected even pace for 60 s. The number of recitations and error rate (incorrect recitation and extended pauses) were logged by the experimenter.

In contrast, a Serial Subtraction (SS) task, an n-Back working memory (2-Back) task and visuospatial Clock task were utilized to target higher-level executive processes. The SS task required participants to verbally count backwards from 100 in 3 s (e.g., “100-97-94”). This task is commonly used in DT studies as a general cognitive task, but is argued to specifically assess executive working memory (Mertens et al., 2006). The experimenter recorded the error rate and the number of subtractions made (participants were instructed to restart at 100 if they reached 0 before the 60 s had elapsed).

An auditory 2-Back task was also employed to assess executive working memory. The participants heard a sequence of nouns from a female voice (Toronto Noun Pool; 16-Bit WAV file; 1411 kbps bit rate; 1500 ms window with 100 ms inter-stimulus delay), presented one at a time, and were asked to respond saying “match” when the current word was a repeat of the word presented two words previously (e.g., “lemon—kitchen—lemon”). The stimulus sequence was different for SCT and DT conditions to control for learning effects. The experimenter logged the participant’s responses for accuracy on the E-Prime presentation laptop.

The Clock task, a visuospatial working memory decision task (adapted from Haggard et al., 2000), presented participants with randomized auditory speech samples announcing a time: e.g., “one-oh-five” (female voice; 16-Bit WAV file; 1411 kbps bit rate; 1000 ms long with a 3000 ms response window from start of stimulus; 500 ms stimulus interval). Participants were required to visualize the time on a clock face and quickly state (“yes” or “no”) whether the hands of the clock were on the same side or not (when the clock is bisected vertically). Again, responses were logged on the E-Prime presentation laptop.

#### Statistical Analyses

Group and task comparisons were made using independent and paired *t*-tests. Due to the multiple comparisons being made, a Bonferroni-corrected alpha was used to avoid a Type 1 error. All statistical analyses were carried out using the IBM SPSS 21 statistical package (SPSS Software, Seattle, WA, USA).

### Results

#### Gait Analysis

A series of independent and paired *t*-tests, (with a Bonferroni correction) were used to compare the groups on each walking trial (SWT and DT conditions) for three gait variables: speed, stride time (STime) and stride time variability (STCV). Only the Clock DT condition revealed a significant difference between the groups, and only for gait speed (*t*_(35)_ = 2.61, *p* = 0.013, *η*^2^ = 0.16). Specifically, the older adults had a significantly slower gait (*M* = 1.23, *SD* = 0.24) than the younger adult group (*M* = 1.41, *SD* = 0.19) while performing the Clock DT (see Table 1). As there were no clear group differences in normal baseline (i.e., single-task) walking gait, correlation analyses between gait and cognitive performances (accuracy and RT) were not conducted.

**Table 1:** **Mean (SD) of speed, stride time (STime) and stride time variability (STCV) on all task for young and older groups**.

	Young			Older		
Task	Speed	STime	STCV%	Speed	STime	STCV%
Normal Walk ST	1.62	0.91	6.69	1.46	0.92	7.02
	(0.20)	(0.08)	(0.76)	(0.19)	(0.08)	(1.07)
Motor DT	1.53	0.93	6.40	1.39	0.95	7.04
	(0.21)	(0.09)	(0.55)	(0.21)	(0.23)	(1.11)
ABC DT	1.46	0.98	7.13	1.32	1.00	6.85
	(0.24)	(0.13)	(1.12)	(0.24)	(0.16)	(1.70)
n-Back DT	1.44	0.97	7.54	1.30	1.00	6.99
	(0.21)	(0.10)	(2.24)	(0.23)	(0.12)	(1.12)
SS DT	1.42	0.98	6.84	1.26	1.04	7.52
	(0.21)	(0.11)	(1.24)	(0.24)	(0.17)	(2.26)
Clock DT	**1.41**	0.98	7.33	**1.23**	1.03	7.25
	**(0.19)**	(0.09)	(1.31)	**(0.24)**	(0.14)	(1.66)

Within each group, the addition of a secondary task while walking resulted in significant decreases in speed from baseline SWT, for all DTs (*p* < 0.001). STime also significantly increased on all DTs (*p* < 0.001), except in the Motor DT condition for the young group (*p* = 0.013). There were no significant differences in STCV between single-task and DT conditions for either group. The DT cost (DTC; %) in speed and STime was calculated to assess the relative change in gait performance on DT conditions, within each group. The DTC was calculated by taking the difference in performance between single-task and DT, dividing it by the single-task performance, and then multiplying this number by 100 (Bock, 2008): $\text{DTC(\%)=}\left(\frac{ST-DT}{ST}\right)*100$.

**Figure 1:**
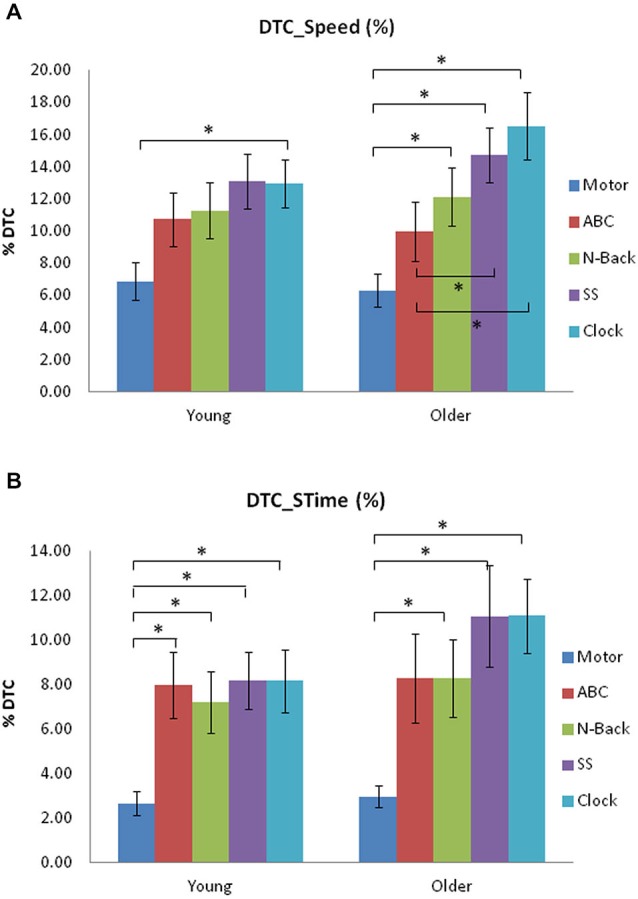
**(A)** Dual-task (DT) Costs (DTC; %) on speed from single-task to each DT for young and older adult groups for the five concurrent tasks used in Experiment 1. **(B)** Dual-task Costs (DTC; %) on stride time (STime) from single-task to each DT for young and older adult groups in Experiment 1. Significant differences are indicated by asterisks: *p < 0.005.

In the younger group, there was a greater DTC for speed during the Clock task compared to the Motor DT (*p* < 0.001, Cohen’s *d* = 1.0; see Figure 1A). In the older group, the DTC for the n-Back (*p* = 0.004, Cohen’s *d* = 0.96,), SS (*p* < 0.001, Cohen’s *d* = 1.45) and Clock task (*p* < 0.001, Cohen’s *d* = 1.51) was significantly greater than the Motor DTC. Furthermore, the DTCs on speed for the SS (*p* = 0.001, Cohen’s *d* = 0.66) and Clock tasks (*p* = 0.001, Cohen’s *d* = 0.82) were also greater than the ABC task’s DTC.

The younger group showed a greater DTC on STime (Figure 1B), for all other DTs in comparison to the Motor DT condition: ABC task (*p* = 0.001, Cohen’s *d* = 1.05); n-Back (*p* = 0.002, Cohen’s *d* = 0.96); SS (*p* < 0.001, Cohen’s *d* = 1.26); Clock task (*p* < 0.001, Cohen’s *d* = 1.16). In the older group, the n-Back (*p* = 0.003, Cohen’s = 1.0), SS (*p* = 0.002, Cohen’s *d* = 1.23), and Clock tasks (*p* < 0.001, Cohen’s *d* = 1.6) elicited a greater DTC on STime than did the Motor task.

These results suggest that the EF-based working memory tasks (n-Back, and Clock tasks) had the largest DT effects on walking gait, which was greater than basic motor/verbal attending and responding (particularly in older adults). This implies an overlap or taxation of concurrent processing of specific higher-level EFs for both the task and walking performance.

#### Cognitive Analysis

Comparing the groups on cognitive performances revealed an age-related decline in accuracy on the Clock task only, for both the SCT and walking DT conditions (ST: *t*_(35)_ = 3.37, *p* = 0.002; DT: *t*_(34)_ = 2.71, *p* = 0.01). Within each group, changes in accuracy and RT from SCT to DT conditions were examined. On the Motor task alone, the younger group revealed a significant increase in RT from SCT (*M* = 587.75, *SD* = 108.20) to DT (*M* = 653.66, *SD* = 100.28) conditions; *t*_(19)_ = 4.72, *p* < 0.001. No other task exhibited DT effects on response performance for either the young or older group. Concurrently, there were no significant differences in the computed cognitive DTC (%) across tasks (the cost on performance in the DT condition was equivalent for all tasks). Given that participants were not told to prioritize either task, the change in gait speed and stride time, but not in cognitive accuracy or RTs suggest that priority was given to the maintenance of the cognitive task performance.

## Experiment 2

### Materials and Methods

A second sample of 20 healthy young adults (50% male; mean age = 21.85 ± 4.58), 13 healthy older adult “non-fallers” (ONF: 46% male; mean age = 70.83 ± 6.66) and 8 older adult “fallers” (OF: 37.5% male; mean age = 63.75 ± 4.43) were recruited for Experiment 2. Older faller profiles were determined by the reporting of at least one fall in the 12 months prior to testing, or in the 6 months following (monitored using monthly fall-calendars which participants completed daily). Falls were defined as “a sudden, unintentional change in position resulting in landing at a lower level (floor, ground or on an object), other than as a consequence of health/medical issues (sudden paralysis, epileptic seizure, medications, or other sickness) or overwhelming external force” (adapted from Tinetti et al., 1997). Falls were considered idiopathic, based on the screened exclusion criteria for relevant diagnoses (as in Experiment 1). Participants completed control tasks and paper-based questionnaires, 2 normal walking gait analysis trials, and 3 seated computer-based tasks with concurrent electrophysiological (EEG) recording of scalp-related potentials. All tasks were completed in one session lasting approximately 2 h (with breaks offered to participants between gait analysis, EEG cap and electrode application, and neuropsychological testing).

There were no significant differences between the young and two older groups in height (*F*_(2,34)_ = 1.64, *p* = 0.21), FES-I score (*F*_(2,38)_ = 0.23, *p* = 0.80), or NART full IQ performance (*F*_(2,38)_ = 0.30, *p* = 0.74). There were no differences in MMSE scores (>28) between OF (*M* = 29.50 ± 0.76) and ONF (*M* = 29.00 ± 1.15); *t*_(19)_ = 1.09, *p* = 0.29. MoCA scores (>23) also did not differ between OF (*M* = 27.13 ± 2.23) and ONF (*M* = 26.39 ± 2.66); *t*_(19)_ = 0.66, *p* = 0.52. A series of one-way ANOVAs with Bonferroni-corrected *post hoc* tests were conducted to compare gait, cognition and the associated ERPs across the 3 groups.

#### Gait Analysis

A straight 15 m walkway on an open, empty corridor was used for walking trials in Experiment 2, which still allowed for extraction of steady pace gait data using an adaptation of the previously outlined algorithm. Again, participants were asked to walk along the walkway 4 times (at SSWS), for 2 trials. Gait data was recorded in the same way, with temporal gait data extracted from the sagittal gyroscope plane. For this experiment, stride time was extracted from the sensor data using the same method employed in Experiment 1 (Greene et al., 2010). Additionally, for Experiment 2, stride length, stride velocity and gait speed were also calculated using a previously validated algorithm (Doheny et al., 2010).

#### Cognitive Tasks

Participants were seated in a quiet, darkened room in front of an E-Prime presentation laptop (Dell Latitude D600 Pentium Laptop, with a 14 inch color monitor). Task-specific instructions were given on-screen and verbally at the start of each task. Where appropriate, response accuracy and reaction times (RT) were recorded automatically in E-Prime. These tasks targeted attentive motor responding, sustained attention/conflict processing and working memory.

The Motor task from Experiment 1 was utilized again as a control task, assessing motor RTs to a single tone stimulus (16-Bit WAV file; 1 s long; 1411 kbps bit rate). The task consisted of 1 test block with 70 trials, presented at a randomly-alternating delay period of 500 ms, 750 ms or 1000 ms, with a maximum response period of 3000 ms.

An n-Back working memory paradigm was again utilized to assess updating/working memory performance (Dobbs and Rule, 1989; Wilhelm et al., 2013). However, a more challenging visual 1-back task was employed which required a response on each trial (and continuous updating of working memory). Visual stimuli were used in this experiment, consisting of gray rectangular placeholders containing the number 1, 2, 3, 4 or nothing (a blank shape) presented on a white background. The stimuli remained on screen for 1800 ms, with a 500 ms fixation between trials. Participants responded to each trial by pressing the numbered key (1, 2, 3 or 4) on the keyboard corresponding to the number presented in the previous trial (1-Back). If the previous trial was blank, no response was required. There was a short practice block of 11 trials (2 blank trials) followed by a test block of 76 trials (69 number trials and 7 blank trials). Again, both accuracy and reaction times were recorded for this task.

A congruent/incongruent judgment Stroop task (word-color stimuli: Stroop, 1935) was administered to assess executive sustained attention, conflict monitoring and response adaptation/switching (Zurrón et al., 2009, 2014). This task visually presented the words “RED”, “GREEN”, “YELLOW” and “BLUE” in either their congruent font color (“RED” in red type) or an incongruent color (“RED” in blue type). There were 2 blocks of 102 trials, each with 90 congruent and 12 incongruent trials presented in random order (with a short break between blocks). The words were presented in the center of the screen on a black background for 1300 ms, with a 350 ms inter-trial blank screen. Participants were required to make a judgment and response on each trial: quickly press the left mouse button when the trial was congruent and the right mouse button for an incongruent trial.

#### EEG Recording and Processing

EEG data were acquired using a BioSemi ActiveTwo system (BioSemi, Amsterdam, Netherlands) with 32 active electrodes applied according to the 10/20 system (American Electrophysiological Association; Crawford et al., 1999), with Signa gel applied to the electrode site to aid conductance. EEG data were sampled at a rate of 1024 Hz, with a pass band filter from 0.16 Hz to 100 Hz. Analogue event triggers were concurrently recorded by the BioSemi system from signals received from the stimulus-presentation laptop. The EEG data were analyzed off-line using Brain Electrical Source Analysis software (BESA; GmbH, Germany). The data were filtered using a 30 Hz high cut-off filter, and referenced to a nasion electrode. Four electro-oculogram (EOG) electrodes (vertical EOG located above and below the left eye; horizontal EOG positioned at the outer canthus of each eye) were used to monitor vertical and horizontal eye movements, which were averaged offline and automatically attenuated using an algorithm in BESA (using an internal model of artifact topographies; Berg and Scherg, 1991; Ille et al., 2002). Following manual removal of remaining movement artifacts, particularly noisy channels were interpolated from surrounding electrodes, or removed, depending on the location of electrode.

Event-related potential (ERP) components were identified and defined based on visual-inspection and previous literature (Mager et al., 2007; Killikelly and Szűcs, 2013; Gajewski and Falkenstein, 2014; Zurrón et al., 2014). ERP segmentations time-locked to stimulus onset were set and averaged in BESA; epoch length −200 to 1,000 ms, with a −200 to 0 ms pre-stimulus baseline correction interval. Grand averages for all participants and participant groups were calculated separately for each task, and mean amplitudes and latencies acted as the dependent variables for all statistical comparisons. Early sensory ERP components (P1, N1, and P2) were preliminarily compared across conditions and groups. Analysis of later EF-related components (N2, P3) on the n-Back and Stroop task were of particular interest, and this analysis was carried out with planned comparisons between the two older groups (ONF and OF).

### Results

#### Gait Analysis

For Experiment 2, five spatio-temporal gait variables were extracted from the kinematic sensor data: velocity, STime, STCV, stride length and stride length variability (SLength and SLCV). There were no significant differences between the 3 groups (young, ONF and OF) on the normal walking gait assessment; *p*-values >0.01 (Bonferroni adjusted). For this reason, normal gait characteristics were not directly correlated with cognitive performances and ERPs. However, comparisons were made between fallers and non-fallers on measures of cognitive performance and the associated ERP waveform components.

#### Cognitive Analysis

Motor task RT, n-Back accuracy and RT, and Stroop accuracy and RT were analyzed between the groups using Bonferroni-corrected one-way or mixed factorial ANOVAs. A main effect of group was found for RTs on the Motor task: *F*_(2,38)_ = 3.56, *p* = 0.038, *η*^2^ = 0.16. *Post hoc* tests using the Bonferroni correction revealed that the younger group responded significantly faster (*M* = 225.64, *SD* = 53.60) than the ONF group (*M* = 363.63, *SD* = 234.31).

An age-related effect between the groups was also observed for 1-Back accuracy: *F*_(2,31)_ = 14.20, *p* < 0.001, *η*^2^ = 0.48, with the younger group performing more accurately (*M* = 98.85, *SD* = 1.43) than both the ONF and OF groups (*M* = 76.75, *SD* = 16.01; *M* = 61.84, *SD* = 31.28). A mixed factorial 3 (groups) × 2 (response accuracy: correct/error) ANOVA showed a significant main effect of response accuracy (Wilks’ Lambda = 0.43, *F*_(1,17)_ = 22.57, *p* < 0.001, *η*^2^ = 0.57), and a response × group interaction effect (Wilks’ Lambda = 0.25, *F*_(2,17)_ = 26.24, *p* < 0.001, $\eta_{p}^{2}$ = 0.76.) whereby the young group had significantly faster reactions times (*M* = 474.1, *SD* = 122.18) when responding correctly than the ONF (*M* = 926.82, *SD* = 179.4) and OF groups (*M* = 1036.36, *SD* = 149.46); *p*-values < 0.001. However, when responding incorrectly (error), the young group had significantly slower reaction times (*M* = 818.7, *SD* = 359.43) than the ONF group alone (*M* = 304.08, *SD* = 206.85): *p* = 0.006, $\eta_{p}^{2}$ = 0.45.

A mixed factorial 3 (groups) × 2 (trial: congruent and incongruent) ANOVA for Stroop task accuracy revealed a significant main effect of trial (Wilks’ Lambda = 0.43, *F*_(1,29)_ = 37.89, *p* < 0.001, $\eta_{p}^{2}$ = 0.57) and a trial × group interaction (Wilks’ Lambda = 0.77, *F*_(2,29)_ = 4.47, *p* = 0.020, $\eta_{p}^{2}$ = 0.24), as well as a main effect of group; *F*_(2,29)_ = 4.27, *p* = 0.24, $\eta_{p}^{2}$ = 0.23). *Post hoc* analyses showed that on incongruent trials, younger adults are significantly more accurate (*M* = 91.18, *SD* = 10.30) than ONF (*M* = 71.67, *SD* = 21.50). These findings were also reflected in RTs: younger adults were significantly faster (*M* = 736.60, *SD* = 82.08) at responding accurately on incongruent trials than the ONF (*M* = 945.25, *SD* = 124.01) and OF groups (*M* = 955.78, *SD* = 134.59); *p*-values < 0.001.

These behavioral results reveal some aging-related effects on cognitive task responses, suggesting that older adults are less accurate and respond more slowly, than young adults on these measures of cognitive performance. However, there were no clear differences between OF and ONF groups.

#### ERP Analyses

The Motor task showed clear N1-P2 auditory evoked-potential (AEP) observable following presentation of the auditory stimulus. One-way between groups ANOVAs were carried out for N1 and P2 mean amplitude and peak latency at left posterior channel P7. N1 peaks were maximal between 95–160 ms post-stimulus. There were no significant differences in N1 amplitude or latency between the young, ONF and OF groups. The P2 component peak was defined between 160–250 ms post-stimulus. There was a large main effect of group for P2 amplitude; *F*_(2,38)_ = 4.9, *p* = 0.013, *η*^2^ = 0.21. *Post hoc* tests revealed a larger mean amplitude for the ONF group (*M* = 4.06, *SD* = 2.59) than the younger group (*M* = 1.92, *SD* = 1.45). However, there were no group differences in P2 latency at P7.

In the n-Back task, three clear ERP components–P1, N2 and P3a–were observed in response to the visual stimulus. One way ANOVAs were carried out between the groups on correct response trials. The P1 was most prominent at occipital electrode O1, occurring between 100 ms and 195 ms. There was no effect of group on P1 amplitude, but there was for peak latency, *F*_(2,31)_ = 7.83, *p* = 0.002, *η*^2^ = 0.36, where P1 latency for the ONF (*M* = 130.60, *SD* = 16.13) and OF group (*M* = 119.63, *SD* = 15.12) occurred significantly earlier than for the younger group (*M* = 155.09, *SD* = 26.11). The N2 peak was maximal between 150 ms and 260 ms post stimulus, and was largest over posterior lead P7. There were no significant differences in mean amplitude or latency between the groups. The P3a was maximal at right posterior PO4 between 230 ms and 430 ms, and also revealed no significant group differences in mean amplitude or latency.

Early P1 and N2 components were observable at posterior sites in response to the visual stimuli on the Stroop task. These were followed by a positive wave in a latency range of 200–385 ms. Repeated measures ANOVAs were conducted to investigate the effect of group (young, ONF, OF) and trial condition (congruent or incongruent) on mean amplitude and latency. The P1 component recorded at occipital electrode O2, occurring between 95 ms and 205 ms, showed no significant main effect of group, or interaction effect on mean amplitude. There was a main effect of group on peak latency however, *F*_(1,32)_ = 5.92, *p* = 0.007, $\eta_{p}^{2}$ = 0.27, wherein the younger group (*M* = 145.43, *SD* = 20.94) showed a significantly later P1 latency than the ONF group (*M* = 126.77, *SD* = 13.87). N2 mean amplitude and peak latency at channel O1 (from 145–210 ms) exhibited a main effect of trial condition (Wilks’ Lambda = 0.82, *F*_(1,32)_ = 7.45, *p* = 0.01, $\eta_{p}^{2}$ = 0.19) and group (*F*_(2,32)_ = 7.45, *p* = 0.002, $\eta_{p}^{2}$ = 0.32). Congruent trials elicited greater N2 amplitude at O1, with the ONF group displaying a significantly larger N2 component on both trial types (*M* = −5.47, *SD* = 3.69) compared to the younger group (*M* = −0.62, *SD* = 3.27). No significant effects were found for N2 latency at O1.

We only identified one later positivity after the N2, presenting at midline Pz and Oz between 200–385 ms post stimulus for both the younger and ONF groups (but not the OF). This component was considered to be an early P3a, based on similar windows and sites reported recently on an equivalent Stroop judgment task (Mager et al., 2007; Zurrón et al., 2009, 2014; Killikelly and Szűcs, 2013). While previous studies have defined the P3a within the window of 300–450 ms post stimulus (Eppinger et al., 2007), more recent investigations of Stroop-related ERPs in young and older adults investigate earlier windows: Killikelly and Szűcs (2013) analyzed an early P3 peak as early as 180–230 ms in young adults, and 250–335 ms in comparable middle aged/older adults.

**Figure 2:**
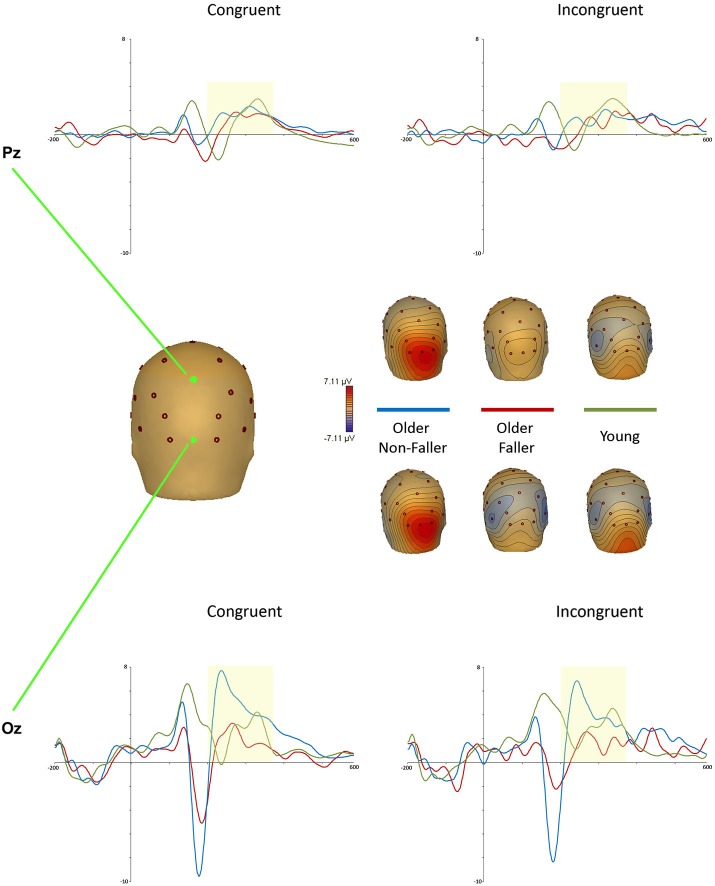
**Stroop task P3a event-related potentials (ERPs) recorded at midline and occipital electrodes Pz (top) and Oz (bottom) for young (green), older non-faller (blue) and older faller (red) groups for congruent and incongruent trials**. Scalp topographies for maximal P3a amplitude on congruent trials (at 229 ms) and incongruent trials (at 234 ms) are shown for each group.

A repeated measures ANOVA revealed a main effect of group for this early P3 peak; *F*_(2,31)_ = 4.88, *p* = 0.014, $\eta_{p}^{2}$ = 0.24. Planned comparisons between the ONF and OF groups revealed a significantly greater peak amplitude for the ONF group on congruent trials at both Pz (OF: *M* = 0.23, *SD* = 0.34; ONF: *M* = 1.39, *SD* = 0.92) and Oz (OF: *M* = 0.36, *SD* = 1.33; ONF: *M* = 3.41, *SD* = 2.69). Peak amplitude was also greater for ONF (*M* = 2.71, *SD* = 2.68) than OF (*M* = 0.18, *SD* = 1.29) on incongruent trials at Oz. Figure 2 illustrates this suggested early P3 (P3a) waveform at Pz and Oz, with Oz scalp topographies at the ONF peak amplitude (congruent: 229 ms; incongruent: 234 ms). There was no main effect for group on P3a latency; *F*_(2,32)_ = 0.42, *p* = 0.66 (corroborating previous findings: West and Alain, 2000).

## Discussion

The two experiments reported here sought to identify the key higher-level cognitive processes, and associated EEG neural activity, underlying walking gait and falls in older adults. Experiment 1 examined walking gait and cognitive performances under single-task and DT conditions in young and older adults. The aim was to investigate the relative effects of varied secondary tasks on gait parameters. Comparisons were drawn directly between non-executive (verbal and motor response) tasks, and three commonly used working memory tasks (n-Back, Serial Subtraction and visuo-spatial Clock tasks). In Experiment 2 we investigated normal walking gait, n-Back and Stroop EF performances and the associated ERPs, in healthy young and older adults, and older adult fallers. We aimed to investigate if there were EF-related ERPs that could act as a marker or indicator of fall-risk in healthy older adults.

### Group Gait Characteristics

We hypothesized differences in quantitative gait speed, stride time and stride time variability during normal (single-task) walking between young and older adults, and older adult fallers and non-fallers. Gait velocity at usual pace has been shown to decline with age (Winter et al., 1990), with speed evidenced as a predictor of falls, identifying fall-risk in community-dwelling fallers (Abellan van Kan et al., 2009; Verghese et al., 2009). Stride time variability and stride length variability have also been shown to predict risk of falls, while cadence and mean stride length have not (Hausdorff et al., 2001; Verghese et al., 2009). However, we were unable to identify any significant differences in gait characteristics between any groups on normal walking trials (single-task baseline) in either of these two experiments. As we recruited healthy community-dwelling older adults who were screened for any neurological, psychological or musculoskeletal impairments, the two main intrinsic risk factors for falls were age and experience of a first fall (WHO report: Todd and Skelton, 2004).

One explanation for this may be that our older adult samples were relatively healthy and active individuals (volunteering from the community and community groups), with comparatively young mean ages (Experiment 1: *M* = 61.88; Experiment 2: ONF *M* = 70.83; OF *M* = 63.75) compared to the samples reported in other studies. However, it is of note that the older fallers had a younger mean age than the older non-fallers. Furthermore, recent literature has also failed to identify age-related differences in spatiotemporal gait variables during normal single-task walking (Terrier and Reynard, 2015). Normal walking trials are also relatively simple to perform, particularly in the controlled environment of the laboratory (a straight, flat, indoor pathway with no distractions or obstructions). Perhaps it is not always possible to identify age-related or fall history differences in gait characteristics when full attention can be dedicated to walking on an easy pathway. This has been shown in a longitudinal prospective study of falls in community-dwelling older adults, where usual walking speed did not predict falls (where age and sex are taken into account; Mirelman et al., 2012). A more challenging pathway for normal walking, or the use of DTs, may be necessary to identify gait differences within healthy, active older adult samples.

### Dual-Task Comparisons

The DT results of Experiment 1 show that specific EF tasks have a greater effect on walking gait than two non-executive secondary tasks. A comparatively greater DTC in speed and stride time was more apparent for the n-Back, SS and Clock executive tasks, and more so in the older adult group, as was hypothesized. However, while the SS and Clock task had a greater effect than both the Motor and ABC tasks, the n-Back task only differed from the Motor task. While the Clock task did appear to have the strongest effect, this could arguably be due to a doubling of executive components (both working memory and visuo-spatial imagining of the clock face). Therefore, this task could have further taxed the EF processing capacities than the other EF tasks, in potentially two sub-domains (and was not utilized in Experiment 2 for this reason).

It is important to note that all DTs effected a change in gait performance, in both young and older adults, relative to single-task walking. This means that divided attention and secondary motor responding play a role in gait performance. These processes are targeted with the low-level ABC recitation and motor tasks in this study, which do not tax higher-level decision making executive control. This novel inclusion of two control non-executive DTs (targeting the verbal/motor functions necessary for responding on the other EF tasks), allows us to identify the additional impact of the decision-making and working memory EF processing. This controlled experimental design allowed us to assess the relative impacts of different secondary tasks, and helped to tackle many of the methodological variability problems reported in the DT literature (Al-Yahya et al., 2011). Further comparably-controlled DT experiments should be undertaken to investigate the relative effects of the secondary tasks which have previously been shown to impact gait in young and older adults.

This study demonstrates that DT changes in gait when completing a secondary task that taxes higher-level cognition are attributable to more than low-level divided attention or motor response processes. These findings specifically show the direct competition for, or taxation of, higher-level EF resources important for walking. These findings are in agreement with previous studies supporting the EF-motor link in relation to gait (Mirelman et al., 2012), and corroborate the idea that older adults may not have adequate cognitive resources. One potential explanation for these effects could be that an underlying executive control system operates as an orchestrating body, allocating resources to and integrating information from the sensory inputs necessary for complex real-world walking. This limited-capacity system may be over-taxed in older adults, where age-related physiological and sensory function decline necessitates compensatory strategies and/or recruitment of additional or alternative cortical regions (Menz et al., 2003; Mirelman et al., 2012).

However, it is of note that the n-Back task which targets EF working memory, did not affect gait more than the ABC control task. This is contrary to what we would have expected, as working memory has been associated with greater DT costs previously, and correlated with fall-risk (Beurskens and Bock, 2012). Similarly, the serial subtraction task had a greater cost than the control ABC task, where the n-Back did not, and can be argued to also tax working memory function. This finding could be due to an overlap in the linguistic characteristics of both the n-Back and ABC tasks, or indicate that the occasional response-criterion in n-Back task used was not as challenging as the SS (and Clock task), which required responses on every trial. Therefore, perhaps this auditory 2-Back task was not challenging enough for young and relatively-young healthy older adults within this paradigm. Yet, in consideration of the findings from Experiment 2, perhaps the working memory sub domain of executive control is not a quintessential process underlying walking gait and fall-risk in older adults.

### Fall Associated ERPs

Due to the absence of group differences in normal walking gait performance, a correlation between seated EF performances and gait metrics (e.g., speed) could not be conducted. Some age-related differences in n-Back and Stroop accuracy and reaction times were observed, which were reflected in some ERP amplitude and latency differences (which is not surprising). However, there were no significant behavioral differences between the older adult groups that could relate to fall-status. This is in contrast to the findings of Buracchio et al. (2011) and Springer et al. (2006), in which EF performance was linked with falls and predictive of DT performance. Our findings could be due to the smaller number of participants in the ONF and older idiopathic OF groups. However, further investigation is needed to assess the validity of cognitive-task screening for falls in older adults without obvious gait impairment (such as the older fallers in this experiment exhibiting comparable normal walking gait performances to young adults).

Despite the lack of differences in behavioral results, there were interesting group differences at the potential early P3a peak on the Stroop task. Only the young and ONF groups exhibited a positive peak after the N2 over parieto-occipital sites, with no further components for the OF group (Figure 2). While it should be noted that the task was largely visual-based, with stimuli which would elicit strong activation over occipital and parietal areas, there is disparity between the positivity reported here and the previous literature defining Stroop P3 components. Age-related P3a components have previously been evident at more fronto-central electrode sites (Fz, Cz), with a more posterior P3b component (not evident here) commonly associated with EF Stroop task processing (Killikelly and Szűcs, 2013; Zurrón et al., 2014). This positivity also presented quite early in comparison to reviews of P3 component windows (Eppinger et al., 2007; Polich, 2007), but was comparable to earlier windows recently reported by Killikelly and Szűcs (2013) in young and older adults.

Despite the somewhat irregular nature in comparison to previous literature, we consider this post-N2 positivity to belong to the EF-related P3 component. Luck (2014) argues that ERP components should not be solely defined by their superficial consequences (latency, scalp distribution and polarity), but rather by the underlying computational operation and neuroanatomy. Considering this, an ERP component may occur at different latencies and sites, but still reflect the associated functional processes. Although this P3a was maximal at midline and occipital electrode sites Pz and Oz, previous functional Magnetic Resonance (fMRI) and EEG studies have posited a frontal generator for the P3a component (McCarthy et al., 1997; Polich, 2007).

Neuroimaging and combined fMRI-ERP studies have evidenced interactive activity in frontal PFC, DLPFC, and ACC areas, and the PPC, in top-down cognitive allocation for the detection of sensory conflict and behavioral response conflict resolution in Stroop conditions (Liston et al., 2006; Kim et al., 2010, 2013; Tang et al., 2013). More recently, focus is on the neural connectivity networks, rather than specific brain regions, underlying Stroop conflict processing. This work demonstrates PPC related higher connectivity within the central executive network (CEN), and lower intra-semantic network (SN) connectivity that positively predicts conflict adaptation via top-down cognitive control (Wang et al., 2015).

A comprehensive review of the P300, P3a and P3b components by Polich (2007) states that P3a generation is considered to occur when adequate attentional focus is applied to the stimulus. After initial sensory processing, attentive stimulus-comparison processing evaluates the stimulus in relation to the previous event in working memory. The potential P3a exhibited in our data by the ONF group was present on both congruent and incongruent trials, likely due to the manual response and congruent/incongruent judgment Stroop task utilized (requiring conflict monitoring and response resolution on each trial). This positivity may reflect frontally-driven attentional responding to the stimulus, or increased top-down CEN connectivity, allowing for the resolution of stimulus-conflict and the appropriate response for congruent and incongruent trials to be determined (Park and Reuter-Lorenz, 2009; Polich, 2007; Wang et al., 2015).

Additionally, this potential P3a, occurring in our fall-free older group, could reflect the adaptive and plastic neural compensation associated with successful aging in healthy older adults (in accordance with the scaffolding theory of aging: Park and Reuter-Lorenz, 2009). It is possible that this difference between the groups underlies, or at least contributes to, their respective fall-status. However, the absence of cognitive performance differences in this study requires further investigation to better elucidate the link between gait, cognitive performance and the scalp-recorded ERPs associated with top-down control in older adult fallers and non-fallers. Possibly a DT condition would have been challenging enough to reveal subtle top-down control impairments in the fallers in line with the ERP findings.

Further analysis focusing on the P3 over frontal areas should be pursued with a larger sample of fallers and non-fallers to specifically examine a potential fall-related lack of frontal attention neural compensation. The possibility of identifying key neuropsychological impairments in fallers which may be reflected in scalp-recorded neural activity not only advances our understanding of falls, but opens an avenue to the application of alternate neurocognitive screening tools in the applied clinical setting. Research investigating cognitive training has recently shown that practice on a Stroop task resulted in increased fMRI recorded neural activity in the ACC, left inferior parietal lobule, and left DLPFC in a modified reading span test targeting attention-switching and conflict resolution between relevant and irrelevant stimuli (Osaka et al., 2012). Better understanding of the key executive processes underlying gait and falls could also lead to potential rehabilitative cognitive training in older adults and high fall-risk clinical samples.

### Conclusions

These two experiments attempted to address the gap of specificity in the existing literature on gait, by exploring the specific nature of the link between cognition and motor output in walking and falls in community-dwelling older adults. The aim was to identify specific neuropsychological executive control tasks which relate to idiopathic falls in older adults. Accessible, wearable and clinically-adaptable wireless sensors and a 32 channel EEG system were used to investigate the precise higher-level EF cognitive processes and related electrophysiology underlying falls. The findings reveal the prominent role of top-down executive control in gait, and may provide tentative evidence of an EF-related ERP component marker of falls. There is a need for further research to comparatively investigate different cognitive and executive processes underlying gait, with advanced investigation of the related neuroelectric activity in older adult fallers. This will require more controlled and comparative DT designs, which could lead to more effective clinical assessment and rehabilitative training techniques. Furthermore, the use of neuroimaging and physiological recording techniques will not only aid in clarifying the cognitive processes at play, but could also translate to the clinical setting for neural screening of fall risk in older adults and clinical samples (e.g., identifying increased fall-risk in stroke after the motor recovery plateau). In conclusion, we provide evidence for a central role of EF in gait and fall risk in older adults, and call for further investigation of ERP activity across fallers and non-faller groups. We argue that advancing our understanding of the higher-level cognitive processes and neural correlates underlying walking gait could significantly ameliorate the mounting healthcare problem of falls.

## Conflict of Interest Statement

The authors declare that the research was conducted in the absence of any commercial or financial relationships that could be construed as a potential conflict of interest.
